# ACL reconstruction with quadriceps tendon graft and press-fit fixation versus quadruple hamstring graft and interference screw fixation – a matched pair analysis after one year follow up

**DOI:** 10.1186/s12891-019-2499-y

**Published:** 2019-03-14

**Authors:** Ralph Akoto, Malte Albers, Maurice Balke, Bertil Bouillon, Jürgen Höher

**Affiliations:** 1 Sports Clinic Cologne at Cologne Merheim Medical Center, Cologne, University of Witten/Herdecke, Ostmerheimerstraße 200, D-51109 Cologne, Germany; 2 Department of Trauma and Orthopedic Surgery, University of Witten/Herdecke, Cologne Merheim Medical Center, Cologne, Germany; 3 Department of Trauma and Reconstructive Surgery, Asklepios Clinic St. Georg, Hamburg, Germany

**Keywords:** ACL reconstruction, Quadriceps tendon, Press-fit fixation

## Abstract

**Background:**

The objective of the study was to compare the results of a primary anterior cruciate ligament reconstruction (ACLR) using the press-fit fixation technique for a quadriceps tendon (QT) graft to a standard quadrupled hamstring (HT) graft with interference screw fixation.

**Methods:**

A retrospective cohort study with a 12-month follow up provided data for 92 patients. Exclusion criteria were accompanying ligament injuries and contralateral ACL injury. Patients who suffered a graft failure, which was defined as a side-to-side difference of > 3 mm, or infection were rated ‘D’ according to the IKDC and excluded from further evaluation. Forty-six patients underwent primary ACLR using the press-fit fixation technique for autologous bone QT graft. These patients were matched in terms of age, gender, accompanying meniscus tear and cartilage injury to 46 patients who underwent standard HT graft with interference screw fixation. Patients were evaluated according to the Lachman test, Pivot-Shift test, IKDC score, Tegner score, Rolimeter measurements, one-leg hop test, thigh circumference and donor side morbidity.

**Results:**

No significant differences in Tegner score (*p* = 0.9), subjective or objective IKDC score (*p* = 0.9;*p* = 0.6), knee stability (Lachman Test p = 0.6; Pivot-Shift Test *p* = 0.4; Side-to-Side Difference *p* = 0.4), functioning testing (One-Leg Hop Test *p* = 0.6; Thigh Circumference *p* = 0.4) or donor side morbidity (*p* = 0.4) were observed at the follow up. The Lachman test was negative for 85% of the QT group and 83% of the HT group. The Pivot Shift Test was negative for 80% of the QT group and 85% of the HT group. The mean side-to-side difference was 1.6 ± 0 .2mm in both groups. The one-leg hop test revealed a collateral-side jumping distance of 96.2 ± 8.5% for the QT group and 95.5 ± 8.5% for the HT group. The thigh circumference of the injured leg was 98.3 ± 3.0% on the uninjured side in the QT group and 99.7 ± 3.0% in the HT group. A knee walking test resulted in no discomfort for 90% of the QT group and 85% of the HT group. The graft failure rate was 7.3% in the QT group and 9.8% in the HT group.

**Conclusion:**

QT grafts fixated using the press-fit technique are a reliable alternative for primary ACL surgery.

## Background

Currently, the autologous hamstring tendon (HT) and the patellar tendon (PT) are the most commonly used grafts for primary anterior cruciate ligament reconstruction (ACLR) [1, 2]. Good clinical results for both grafts have been reported in the literature [3]. However, recent literature has shown that these grafts have some disadvantages. In a considerable proportion of patients, harvesting the patellar tendon leads to discomfort on the donor side [4, 5]. In addition, recent literature has shown that HT grafts have a higher risk of failure than do PT grafts [6–9]. Quadriceps tendon (QT) grafts are currently considered only a second choice graft, although its good biomechanical and biological characteristics has been demonstrated in several studies [10, 11]. Clinical studies comparing QT and PT grafts showed comparable clinical results but lower donor side morbidity for the QT graft [12–15]. For these reasons, QT grafts have been proposed as a promising alternative to the common grafts in ACLR surgery.

To our knowledge, until now, four studies have been published, which compared QT and HT grafts in primary ACLR [16–20], However results were inconsistent, therefore more data directly comparing this two grafts is necessary.

Press-fit fixation is an alternative to conventional interference screw fixation. Biomechanical studies demonstrated adequate primary stability with ultimate load to failure pull forces at least equal to published results for interference screws [21, 22]. Excellent clinical results for different grafts types fixated in press-fit technique were reported [23–27].

In comparison to interference screw fixation press-fit fixation techniques showed less bone tunnel widening [28, 29]. To our experience in case revision surgery the bone tunnel management is less difficult.

### Hypothesis

Primary ACLR using the press-fit technique to fixate QT grafts achieves comparable results to interference screw fixation and standard quadrupled HT grafts.

## Methods

Between December 2010 and March 2013, 120 patients with primary ACL insufficiency were enrolled in the study. Patients were included in this study if they met the following criteria: (1) older than 18 years of age, (2) primary ACL surgery, (3) no concomitant ligament injury, (4) unilateral ACL injury, (5) no previous surgery on the effected knee, (6), no chondral lesion worse than Outerbridge grade 2, (8) clinically and MRI confirmed ACL rupture. According to the methodology of a previously published study, 60 patients underwent ACLR with the press-fit technique used to fixate QT grafts [28]. Sixty patients who underwent an ACLR with interference screw fixation (PLDLLA; MEGAFIX®; Karl Storz AG) with a fourfold semitendinosus graft were selected as control group for a comparative matched-pair analysis. Matched pair criteria (age, presence of an accompanying meniscus tear or cartilage injury, additional meniscus or cartilage surgery), the surgeon performing the operation (senior author J.H.), graft selection (patients were free to choose the desired graft), the surgical techniques and the rehabilitation protocol were identical to the studies published in the apron [23, 28] Patients with accompanying ligament injuries (collateral ligaments or posterior cruciate ligaments) or contralateral ACL injury were excluded. Patients who suffered a graft failure or infection were rated as “D” according to the International Knee Documentation Committee (IKDC) criteria and excluded from further evaluation.

### Surgical technique

The surgical technique was described in detail in a study published in advance [28] .

### Postoperative evaluation

Follow-up examinations were performed for the QT group 14 ± 1.4 months after ACLR and for the HT group at 13.7 ± 2.5 months after ACLR. The duration of the graft surgery was recorded for each patient.

Subjective and objective IKDC scores and Tegner activity level scores were measured. Knee stability was assessed with the Lachman test (possible results: negative, 1+, 2+ and 3+), Pivot-shift Test (possible results: negative, (+) glide, + and ++) and a Rolimeter® (Aircast Europa GmbH, Neubeuern, Germany) to perform instrumental laxity measurement. Graft failure was indicated by a side-to-side difference of more than 3 mm in the instrumental measurement.

In addition, a one-leg-hop test was performed and the thigh circumference 20 cm above the joint line was measured. To describe donor side morbidity after graft harvesting, a knee walking test with a four-point scoring system described by Kartus et al. was used [30].

### Statistics

Statistical analysis was performed using the SPSS 24.0 software package (SPSS, Inc. Chicago, Illinois). A paired t-test was used to evaluate parametric data, and non-parametric data was evaluated using the Wilcoxon test. A *p*-value of < 0.05 was assumed to be statistically significant. A post hoc power analysis was performed with G*Power 3.1.9.3 to assess the validity of the number of patients, based on the comparison of the mean Tegner score at the time of follow up and all other variables. With these effect sizes, an alpha of 0.05, and sample size of 41, a power ranging from 0.05 and 0.95 was calculated (0.05 for Tegner score and subjective IKDC, 0.08 for One-Leg Hop Test, 0.1 for donor side morbidity and objective IKDC, 0.7 for postoperative side-to-side difference, for 0.83 for thigh circumference, 0.84 for Pivot-Shift Test, 0.87 for Lachman Test, 0.95 for Duration of surgery).

## Results

Seven patients who suffered a graft failure (three in the QT group, four in the HT group) and three patients who had an infection (one in the QT group, two in the HT group) were rated as “D” according to the IKDC criteria, and the matched pairs were excluded from follow-up examinations. In addition, the matched pairs for three cases of contralateral ACL rupture (one in the QT group, two in the HT group) and nine cases (three in the QT group, six in the HT group) that were lost to follow up were excluded. Three new matched pairs were formed from six patients (three in the QT group, three in the HT group) whose matched partner was excluded. Eighty-two patients (41 in the QT group, 41 in the HT group) were available for the one-year follow-up examination. No statistical differences were found between the two groups in terms of graft failure, infection, contralateral ACL rupture or accompanying meniscus and cartilage injuries. The QT group underwent statistically significantly longer operations compared to the HT group.

Table 1 shows the demographic data from the baseline, accompanying injuries, complications and duration of operation for both groups.

**Table 1:** Demographic data, accompanying injuries and complications of the included patients

	QT Group (*N* = 41)	HT Group (*N* = 41)	*P*-value
Male	32	32	
Mean age (years)	29 ± 10	28 ± 10	0.495
Accompanying injuries			
Meniscus (medial/lateral)	8 (6/2)	8 (5/3)	0.485
Chondral lesions	3	2	0.486
Complications			
Graft failure	3 (7.3%)	4 (9.8%)	0.694
Contralateral ACL rupture	1	2	0.644
Infection	1	2	0.556
Mean and SD [mm]	4.3 ± 2.2	4.5 ± 1.7	0.8
Duration of surgery (min)	96.6 ± 12.0	86.9 ± 11.8	< 0.01*

*Paired t-tests indicate a statistically significant difference in the duration of surgery. No statistically significant differences were found in terms of demographic data, accompanying injuries or complications. *QT* = Quadriceps tendon; *HT* = Hamstring tendon

### Functional scores

No significant differences were found between the groups with regards to functional scores. The mean Tegner score at the time of follow-up was 7.6 ± 1.8 for both groups (*p* = 0.9).

**Fig. 1:**
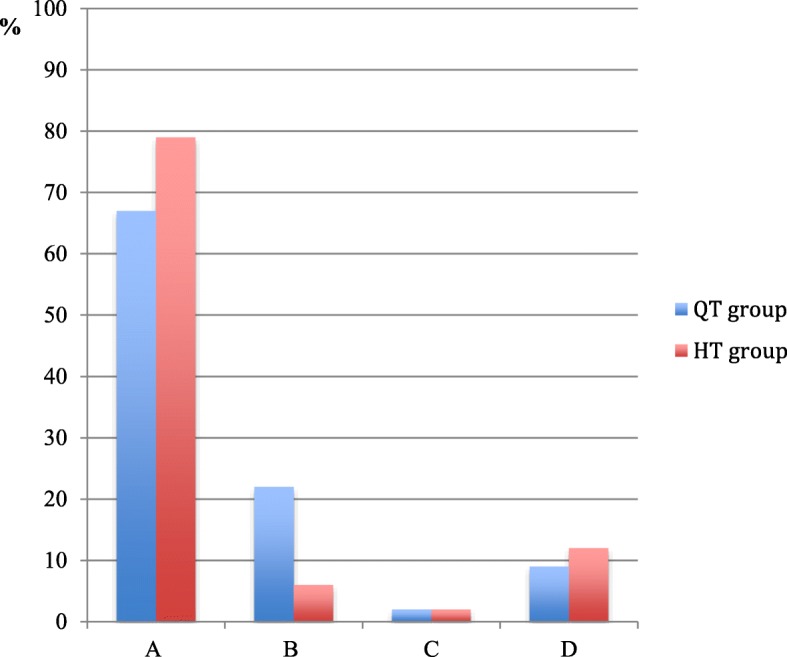
Objective IKDC scores in percent of the QT group and the HT group at the time of follow up. No significant differences were found between the groups. **a** = normal; **b** = nearly normal; **c** = abnormal; **d** = severely abnormal

The mean subjective IKDC scores were 86.4 ± 14.2 in the QT group and 86.7 ± 10.9 in the HT group (*p* = 0.9). The objective IKDC scores are shown in Fig. 1.

### Postoperative knee stability

No significant difference between the groups was found regarding postoperative knee stability, measured by the Lachman test, Pivot-Shift test or instrumental measurement (Tables 2 and 3).

**Table 2:** Postoperative knee stability (Lachman test, Pivot-Shift test)

N		QT group (%)	HT group (%)	*p*-value
		41	41	
Lachman Test				0.6
	negative	35(85)	34(83)	
	+	6(15)	7(17)	
	++	0	0	
Pivot-Shift Test				0.4
	negative	33(80)	35(85)	
	glide	2(5)	4(10)	
	+	6 (15)	2(5)	
	++	0	0	

Wilcoxon test indicates no statistically significant difference between the QT- and the HT group in objective knee stability (Lachman test, Pivot-Shift test) between the QT group and the HT group; *QT* = Quadriceps tendon; *HT* = Hamstring tendon

**Table 3:** Postoperative side-to-side difference (instrumental measurement, Rolimeter®)

N	QT group	HT group	*p*-value
	41	41	
Mean and SD [mm]	1.6 ± 0.2	1.6 ± 0.2	0.8

Paired t-test indicates no statistically significant difference between the QT- and the HT group in objective knee stability (instrumental measurement, Rolimeter®) between the QT group and the HT group. *QT* = Quadriceps tendon; *HT* = Hamstring tendon

Functional tests (one-leg hop test and thigh circumference).

No statistical differences between the QT- and the HT group were found at the follow up regarding one-leg hop test results and thigh circumference (Table 4).

**Table 4:** One-leg hop test and thigh circumference

N	QT group	HT group	*p*-value
	41	41	
One-Leg Hop Test^a^	96.2 ± 8.5	95.5 ± 8.5	0.8
Thigh Circumference^b^	98.3 ± 3.0	99.7 ± 3.0	0.2

^a,b^The difference between the injured leg and uninjured leg was given as a per cent. Paired t-test indicated no statistically significant difference between the QT- and the HT group. *QT* = Quadriceps tendon; *HT* = Hamstring tendon

### Donor side morbidity

**Fig. 2:**
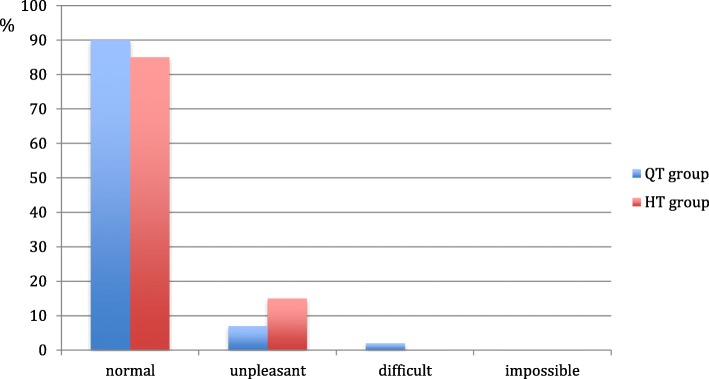
Knee walking test of the QT group and the HT group at the time of follow up. The patients report their subjective discomfort in knee walking in the levels: no problems, minor problems, major problems or unable to perform the test. No significant differences were found between the groups

In total, 90% of the QT group and 85% of the HT group had no discomfort when asked to walk on their knees at the follow up. No statistically significant differences were found between the groups (*p* = 0.4) (Fig. 2).

## Discussion

The most important finding of this study is that a QT graft with press-fit fixation achieved comparable clinical and functional results as a standard HT graft with interference screw fixation.

Few previous studies have directly compared the clinical data of primary ACLR with QT grafts to that of ACLR with HT grafts, but the results are inconsistent (Table 5) [16–19].

**Table 5:** Clinical studies that directly compare ACLR with QT vs. HT

Author	Number of patients and Surgical Technique, FU Drilling Technique Femoral/Tibial Fixation	Main Results	Graft Failure
Runer et al. [16] 2017	40 SB QT-PB vs. 40 SB HT; 2 years Both: AM; button/Bio-IF screw + button	No difference in Lyholm, Tegner and VAS scores or knee pain; no postoperative knee laxity data	1 HT 0 QT
Cavaignac et al. [17] 2017	45 QT-BT vs. 41 SB HT, 3.6 years Both: outside in, Bio-IF screw/Bio-IF screw	No difference in Tegner and IKDC scores; Sig. better Lysholm and KOOS scores for QT; SSD QT-HT 1.1 mm vs. 3.6 mm	1 QT 2 HT
Lee et al. [18]2016	48 SB QT-PB vs. 48 dB HT, 2 years QT: TT, metal IF screw/Bio-IF screw HT: AM; button/Bio-IF screw	No difference Lysholm, Tegenr, IKDC, SSD QT 2 .1mm; HT 1 .9mm; no difference in anterior knee pain and extensor muscle strength recovery, better flexor muscle strength recovery for QT	No data
Sofu et al. [19] 2017	23 SB QT-PB vs. 21 SB HT; both: TT, 3 years QT: Metal-IF screw/Bio-IF screw HT: Transfemural fixation/Bio-IF screw	SSD: QT-HT 2.8 vs. 1.1, QT 52% > 3 mm, HT 9% > 3 mm Symptoms of instability: 21% QT, 0% HT	No data

*FU* = Follow up; *SB* = Single bundle; *DB* = Double bundle; *QT* = Quadriceps tendon, *PB* = Patellar bone; *HT* = Hamstring tendon; *AM* = Anteromedial portal drilling technique; *TT* = Transtibial drilling technique; Bio-IF-screw = bioresorbable interference screw; *SSD* = Side-to-side difference (KT1000/Rolimeter); *VAS* = Visual analogue scale; *KOOS* = Knee injury and osteoarthritis outcome score; *AM* = Anteromedial drilling technique, *QT-BT* = Quadriceps bone-tendon graft

Cavaignac et al. and Lee et al. reported the results of cohort studies comparing bone tendon QT graft and HT graft with 3.6 and 2 years follow up, respectively [17, 18]. Both authors achieved comparable results to our study regarding knee stability at the time of follow up (QT graft at FU: instrumental measurement 1.1 ± 0.9 mm vs. 2.1 ± 1.9 mm vs. 1.6 ± 0.2 mm side-to-side difference; 93% vs. 67% vs. 80% negative pivot shift). Sofu et al. did not achieve similar results [19]. In their study, 21% of QT graft recipients suffered from persistent symptoms of knee instability, and only 48% had side-to-side difference of < 3 mm. Sotu et al. justified these results, stating that the knee extensor was weakened, which increased the biomechanical stress of the graft and may have led to early graft failure. In our study, graft failure was defined as a side-to-side difference of > 3 mm. Our definition of graft failure was stricter than that of Sofu et al. and other studies comparing QT and HT grafts. In our study, the failure rate of QT grafts was 7.3%, which is much lower than that obtained by Sofu et al. and comparable to the results of Cavaignac et al., who reported 5 patients (11.4%) with a side-to-side difference of < 3 mm at the follow up.

Concerning the results of the functional tests of this study, in the QT group, the participants achieved an average of 96% of hop distance with the injured legs compared to the uninjured legs in the one-leg hop test, and a mean calf circumference of 98% for the injured- compared to the uninjured legs at the follow up. These results align with the isokinetic strength tests of Lee et al., who reported no difference in knee extensor strength between QT and HT at follow up [18]. The results of Lee et al. as well as our findings shows that harvesting QTs does not led to biomechanical weakening of the knee extensor mechanism.

Studies examining donor side morbidity after harvesting grafts have found that QT grafts lead to significantly less anterior knee pain than do PT grafts [12–14, 31, 32]. To our knowledge, until now, the only authors to directly compare of donor side morbidity after harvesting QT and HT grafts were Runer et al., Cavaignac et al. and Lee et al. Like our study, all three studies revealed no significant difference in anterior knee pain between the two types of grafts [16–18].

In our study, grafts were fixated using the press-fit technique. Several studies have described the use of press-fit fixation techniques for ACL surgery [24–26, 33, 34]. In biomechanical investigations, press-fit fixation has shown sufficient strength, comparable to interference screw fixation [21]. For the tibial tunnel, we performed a hybrid technique involving press-fit fixation near the joint line and distal cortial suture fixation. In another study examining a subgroup of this study population, we show that this fixation technique leads to less bone tunnel dilation than interference screw fixation in the tibial tunnel [28].

Our results show that press-fit fixation for QT grafts could be an alternative to conventional fixation techniques. It is advantageous as it both saves fixation material, resulting in lower cost, and involves less bone tunnel enlargement, which could aid revision surgery.

### Limitations

This study had several limitations. First, each group received a different graft fixation technique which may have influenced the results. Nevertheless, previous studies have shown that press-fit fixation produces comparable results to conventional fixation techniques [24, 25, 33]. Second, some authors have stated that a follow up of 1 year after ACLR might be too short to evaluate postoperative outcomes. However, primary graft healing is complete after 12 months [35]. Third, the patients were free to choose the type of graft they received. This could have produced bias concerning the outcomes of our study. However, to produce comparable results, each patient in the QT group was matched to a patient in the HT group using the aforementioned criteria, all patients were the same gender and all procedures were done by one surgeon.

## Conclusion

QT graft fixated with the press-fit technique achieved good results comparable to those of a standard HT graft with interference screw fixation. Thus, it is reliable alternative for primary ACL surgery.

## Abbreviations

ACLR: Anterior cruciate ligament reconstruction
HT: Hamstring tendon
IKDC: International Knee Documentation Committee
PT: Patellar tendon
QT: Quadriceps tendon
